# The Relationship between Muscle Fiber Type-Specific PGC-1α Content and Mitochondrial Content Varies between Rodent Models and Humans

**DOI:** 10.1371/journal.pone.0103044

**Published:** 2014-08-14

**Authors:** Gilles Gouspillou, Nicolas Sgarioto, Brandon Norris, Sébastien Barbat-Artigas, Mylène Aubertin-Leheudre, Jose A. Morais, Yan Burelle, Tanja Taivassalo, Russell T. Hepple

**Affiliations:** 1 Department of Critical Care, McGill University Health Centre and Department of Medicine, McGill University, Montréal, Québec, Canada; 2 Département de kinanthropologie, Faculté des sciences, Université du Québec À Montréal, Montréal, Québec, Canada; 3 Department of Kinesiology and Physical Education, McGill University, Montréal, Québec, Canada; 4 Division of Geriatric Medicine, McGill University Health Centre, McGill University, Montreal, Québec, Canada; 5 Faculty of Pharmacy, Université de Montréal, Montréal, Québec, Canada; Charité Universitätsmedizin Berlin, NeuroCure Clinical Research Center, Germany

## Abstract

PGC-1α regulates critical processes in muscle physiology, including mitochondrial biogenesis, lipid metabolism and angiogenesis. Furthermore, PGC-1α was suggested as an important regulator of fiber type determination. However, whether a muscle fiber type-specific PGC-1α content exists, whether PGC-1α content relates to basal levels of mitochondrial content, and whether such relationships are preserved between humans and classically used rodent models are all questions that have been either poorly addressed or never investigated. To address these issues, we investigated the fiber type-specific content of PGC-1α and its relationship to basal mitochondrial content in mouse, rat and human muscles using in situ immunolabeling and histochemical methods on muscle serial cross-sections. Whereas type IIa fibers exhibited the highest PGC-1α in all three species, other fiber types displayed a hierarchy of type IIx>I>IIb in mouse, type I = IIx> IIb in rat, and type IIx>I in human. In terms of mitochondrial content, we observed a hierarchy of IIa>IIx>I>IIb in mouse, IIa >I>IIx> IIb in rat, and I>IIa> IIx in human skeletal muscle. We also found in rat skeletal muscle that type I fibers displayed the highest capillarization followed by type IIa >IIx>IIb. Finally, we found in human skeletal muscle that type I fibers display the highest lipid content, followed by type IIa>IIx. Altogether, our results reveal that (i) the fiber type-specific PGC-1α and mitochondrial contents were only matched in mouse, (ii) the patterns of PGC-1α and mitochondrial contents observed in mice and rats do not correspond to that seen in humans in several respects, and (iii) the classical phenotypes thought to be regulated by PGC-1α do not vary exclusively as a function of PGC-1α content in rat and human muscles.

## Introduction

PGC-1α is a coactivator of transcription involved in multiple aspects of skeletal muscle physiology. Amongst the best-characterized roles of PGC-1α is its control over mitochondrial biogenesis. Muscle-specific overexpression of PGC-1α results in the activation of genes regulating mitochondrial oxidative metabolism [1] and increases mitochondrial content in both cardiac and skeletal muscles [2]–[5]. In contrast, muscle-specific Knock Out (MKO) of PGC-1α was shown to reduce mitochondrial gene expression and mitochondrial content in mouse skeletal muscle [6]. Furthermore, in PGC-1α MKO mice it was recently shown that PGC-1α plays a critical role in endurance training-induced mitochondrial biogenesis [7], although this latter finding has been recently challenged [8].

In addition to controlling mitochondrial biogenesis, PGC-1α plays a crucial role in the regulation of angiogenesis where muscle-specific overexpression of PGC-1α was shown to increase the capillary density in mouse skeletal muscle [9]. Furthermore, muscle-specific overexpression of PGC-1α speeds the recovery of muscle blood flow following the ligation and ablation of the femoral artery, while muscle blood flow recovery is severely blunted in PGC-1α^−/−^ animals following the same surgery [9].

Recently, it was also suggested that PGC-1α regulates lipid droplet formation in skeletal muscle since PGC-1α overexpressing animals display an increase in intramyocellular lipid droplets [10], a phenomenon that was also observed in cultured human skeletal muscle cells overexpressing PGC-1α [11].

PGC-1α has also been implicated in fiber type determination, where PGC-1α overexpression in mouse skeletal muscle leads to an increase in type I proportion fibers in the plantaris muscle [1]. In addition, oxidative muscles were shown to contain more PGC-1α than glycolytic muscles [1]. These results led to the notion that PGC-1α drives the formation of type I fibers [1] and to widespread implication that type I fibers have the highest PGC-1α content.

Altogether, this collection of data strongly suggests that PGC-1α plays a central role in coordinating both metabolic and contractile profiles of skeletal muscle fibers. However, to date, the fiber type-specific content of PGC-1α remains unclear and its relationship with basal mitochondrial content has never been previously investigated in a direct way. In addition, whether the relationships between PGC-1α content and mitochondrial content observed at the individual fiber type level in typically used rodent models are consistent with what is seen in humans has never been investigated. Given the utility of rodent models in establishing our mechanistic understanding of the role of PGC-1α in skeletal muscle, such information is crucial to inform future investigations and particularly clinical translation. To address these issues, the present study investigated the fiber type-specific PGC-1α and mitochondrial contents in mouse, rat and human skeletal muscles. The relationship between the fiber type-specific PGC-1α content and capillarization was also investigated in rat skeletal muscle. Finally, the relationship between fiber type-specific PGC-1α content and lipid droplet content in human muscle was investigated in human muscle. We hypothesized that differences in PGC-1α content across fiber types should relate to differences in mitochondrial content regardless of species. Furthermore, we hypothesized that fiber type-specific PGC-1α content examined in rat muscle should relate to fiber type differences in degree of capillarization. Finally, we hypothesized that fiber type-specific PGC-1α content examined in human muscle should relate to fiber type differences in lipid droplet content.

## Materials and Methods

### Ethics statement

Animals: All procedures were approved by the University of Calgary Animal Care Committee (BI09R-11; rats) or the McGill Animal Care Committee (2012-7189; mice).

Humans: All participants were fully informed about the nature, goal, procedures, and risks of the study and gave their written informed consent. All procedures, including the consent procedure, were approved by the “Comité institutionnel d’éthique de la recherche avec des êtres humains de l’UQAM” (Approval number: 709987)”.

### Animals and Tissue Harvest

Adult male C57/Bl6 mice (8- to 12-wk-old) were kindly provided by the Dr. Yan Burelle from the Université de Montréal. Adult Male Fisher 344 X Brown Norway F1 hybrid rats (8- to 10-mo-old) were obtained from the National Institute on Aging (NIA; Baltimore, MD). Animals were euthanized with pentobarbital. For mice, the gastrocnemius (Gas) was removed. For rats, both the plantaris (PL) and soleus (SOL) muscles were collected. Once removed, muscles were dissected free of fat. A slice through the entire midbelly of each muscle was mounted on cork in optimal cutting temperature compound and frozen in liquid isopentane cooled in liquid nitrogen. Samples were stored at −80°C until use.

### Subjects and Muscle Tissue Harvest

Muscle biopsy samples of the right vastus lateralis (VL) muscle were obtained from six young adult men (23.8 yo ±1.3; mean ± SEM) by standard Bergström needle technique as described previously [12]. Solid pieces of muscle (approx. 40 mg) were mounted with the muscle fibers in transverse orientation on specially engineered plastic blocs in tragacanth gum and frozen in liquid isopentane cooled in liquid nitrogen. Samples were stored at −80°C until use. All procedures were approved by “Comité institutionnel d’éthique de la recherche avec des êtres humains de l’UQAM” (Approval number: 709987).

### Western Blotting

PGC-1α content was determined in muscle homogenates prepared from mouse gastrocnemius muscles, rat soleus and plantaris muscles, human vastus lateralis muscles, as well as a mixed skeletal muscle homogenate from PGC-1α^−/−^ mice (sample kindly provided by the Dr. Julie St-Pierre, McGill University). Approximately 40 to 50 mg of the each muscle was homogenized in 10 volumes of an extraction buffer composed of Tris base 50 mM, NaCl 150 mM, Triton X-100 1%, Sodium deoxycolate 0.5%, SDS 0.1% and 10 µl/ml of a protease inhibitor cocktail (Sigma P8340). The homogenate was centrifuged at 15,000 *g* for 15 min at 4°C. Protein content in the supernatant was determined using the Bradford method [13].

Aliquots of supernatant were mixed with Laemli buffer and subsequently boiled at 95°C for 5 min. Proteins (approx.15 µg for mouse gastrocnemius muscle, rat soleus and plantaris muscles, 30 µg for human vastus lateralis muscle and 40 µg for muscles from PGC-1α^−/−^ mice) were loaded onto 8% gels, electrophoresed by SDS-PAGE and then transferred to polyvinylidene fluoride membranes (Life Sciences). Membranes were incubated for 1 h at room temperature in a blocking buffer composed of 5% (w/v) non-fat dried milk in Tris-buffered saline containing 0.1% Tween 20 (TBS-T) and then incubated overnight at 4°C with a rabbit polyclonal anti- PGC-1α (Millipore, Ab3242, 1∶50) and a rabbit polyclonal anti- β-Tubulin (Abcam, Ab6046, 1∶500) diluted in blocking buffer. Membranes were washed 6 times for 5 min each in TBS-T and subsequently incubated with HRP-conjugated secondary antibodies (Abcam Ab6721 and Ab6728) diluted in blocking buffer 1 h at room temperature. PGC-1α signals were detected using enhanced chemiluminescence substrate (Thermo Scientific, 24080) and analyzed using ImageJ (NIH).

### Skeletal Muscle Processing

#### Sectioning

Eight micron thick serial cross-sections were cut in a cryostat at −18°C and mounted on lysine coated slides (Superforst) to dertemine fiber type, PGC-1α mice content, mitochondrial content and capillarization.

#### *In situ* determination of fiber type

For animals, the two first sections of each series were immunolabeled for the different myosin heavy chains (MHC) using a previously described method [14]. Briefly, the first cross-sections of each animal sample were used to immunolabel for MHC type I, IIa and IIb. These cross-sections were first allowed to reach room temperature and rehydrated with PBS (pH 7.2). These sections were then blocked using goat serum (10% in PBS) and incubated for 1 hour at room temperature with the following primary antibody cocktail: mouse IgG2b monoclonal anti-MHC type I (BA-F8, 1∶25), mouse IgG1 monoclonal anti-MHC type IIa (SC-71, 1∶200), mouse IgM monoclonal anti-MHC type IIb (BF-F3, 1∶200) and rabbit IgG polyclonal anti-laminin (Sigma L9393, 1∶750). Muscle cross-sections were then washed three times in PBS before being incubated for 1 hour at room temperature with the following secondary antibody cocktail: Alexa Fluor 350 IgG2b (y2b) goat anti-mouse (Invitrogen, A-21140, 1∶500), Alexa Fluor 594 IgG1 (y1) Goat anti-mouse (Invitrogen, A-21125, 1∶100), Alexa Fluor 488 IgM goat anti-mouse (Invitrogen, A-21042, 1∶500) and Alexa Fluor 488 IgG goat anti-rabbit (A-11008, 1∶500). Muscle cross-sections were then washed three times in PBS and slides were then cover slipped using Prolong Gold (Invitrogen, P36930) as mounting medium.

Identical procedures were employed for the cross-section used to immunolabel for MHC type IIx, except the primary antibody cocktail which was comprised of a mouse IgM monoclonal anti-type 2x MHC (6H1, 1∶25) and a rabbit IgG polyclonal anti-laminin, and the secondary antibody cocktail that was comprised of Alexa Fluor 488 IgM goat anti-mouse and Alexa Fluor 488 IgG goat anti-rabbit.

As healthy human muscle expresses only 3 different types of MHC at the protein level (type I, IIa, IIx) [15], human muscle cross-sections were immunolabelled using the protocol described previously at the exception of the primary antibody cocktail that was composed of a mouse IgG2b monoclonal anti-MHC type I (BA-F8, 1∶25), mouse IgG1 monoclonal anti-MHC type IIa (SC-71, 1∶200), mouse IgM monoclonal anti-type 2x MHC (6H1, 1∶25) and a rabbit IgG polyclonal anti-laminin. All primary antibody targeting MHCs were purchased from the Developmental Studies Hybridoma Bank (DSHB, University of Iowa, IA).

#### *In situ* determination of PGC-1α content

For each sample, the muscle cross-section that was cut immediately following those used for MHC immunolabeling was used to determine PGC-1α content *in situ*. Sections were first allowed to reach room and were fixed in acetone at 4°C for 15 min. Samples were then washed for 5 min in PBS (pH 7.4) at 4°C before being incubated for 15 min in a permeabilization solution 0.1% Triton X-100 in PBS) at 4°C. Slides were then washed 3 times in PBS, before being incubated at 4°C in a blocking solution (10% goat serum in PBS) for 15 min at room temperature. Slides were then incubated overnight at 4°C with a rabbit IgG polyclonal anti- PGC-1α (Millipore, Ab3242, 1∶50). For mouse muscle cross-sections a mouse IgG monoclonal anti-dystrophin (Sigma, D8168, 1∶100) was also applied. The following day, slides were washed 3 times in PBS at 4°C before being incubated for 90 minutes at room temperature with an Alexa Fluor 488 IgG goat anti-rabbit (A-11008, 1∶500) and an Alexa Fluor 647 goat anti-mouse IgG, (Invitrogen, A-21235, 1∶100) for mouse muscle cross-sections. Cross-sections were washed 3 times in PBS at 4°C before a 10 min incubation in a PBS solution containing DAPI (Invitrogen, D1306, [300 nm]) at 4°C. Slides were then washed 3 times in PBS and finally cover slipped using Prolong Gold (Invitrogen, P36930) as mounting medium.

#### *In situ* determination of mitochondrial content using the Succinate dehydrogenase stain

The serial muscle cross-section cut immediately after the one used for PGC-1α immunolabeling was used to determine mitochondrial content *in situ*. Sections were stained for succinate dehydrogenase (SDH, complex II of the respiratory chain) activity as follows: Sections were first allowed to reach room temperature and were rehydrated with PBS (pH 7.2). Sections were then incubated in a solution containing Nitroblue tetrazolium (1.5 mM), Sodium succinate (130 mM), Phenazine methosulphate (0.2 mM) and Sodium azide (0.1 mM) for 20 min for rat and mouse sections or 45 min for human sections. Cross-sections were then washed 3 times in PBS, dehydrated in 75% (30 s), 90% (30 s) and 100% (10 min) ethanol and cover-slipped using an aqueous mounting medium (Vector Labs, VectaMount AQ Medium, H-5501). All samples for each species were processed at the time and using the same incubation solution, ensuring that all samples underwent the exact same conditions.

#### *In situ* determination of mitochondrial content using immunolabeling for Translocase of the outer membrane 20 (TOM20) and Voltage-dependent anion channel (VDAC)

Procedures identical to those described for the PGC-1α immunolabeling were used to immunolabel for VDAC and TOM20, at the exception of the fixation step for the TOM20 stain that was performed in 2% paraformaldehyde for 30 min. In addition of being labeled for either VDAC or TOM20, sections were also labeled for laminin (for the VDAC stain) or dystrophin (for the TOM20 stain). The primary antibodies used for these experiments were the following: a mouse monoclonal IgG anti-VDAC1 antibody (Mitosciences, MSA03) or a rabbit polyclonal IgG anti-TOM20 antibody (Santa-Cruz, sc-11415).

#### *In situ* determination of muscle capillarization

To assess muscle capillarization, the Lead ATPase stain method developed by Rosenblatt et al [16] was applied to the muscle cross-section serial to the one used for mitochondrial content determination. Briefly, as we have done previously in rat muscle [17], the sections were first fixed for 5 min in a Guth and Samaha fixative [18] at 4°C, rinsed in distilled water and then incubated for 1 h at 37°C in a Lead ATPase staining medium to stain for capillaries [16]. Sections were then washed in distilled water and developed for 1 min in 2% amonium sulfide. Sections were finally washed in distilled water and cover-slipped using an aqueous mounting medium (Vector Labs, VectaMount AQ Medium, H-5501).

#### *In situ* determination of lipid content

To determine the intramyocellular lipid content, the Oil Red O stain method (Oil Red O being a fat-soluble and fluorescent dye) was used [19]. Briefly, muscle cross sections were first allowed to reach room temperature and then fixed in 10% formalin (Sigma-Aldrich, HT501128) for 5 minutes. Sections were then washed 3 times 1 minute in distilled water before being placed in propylene glycol for 2 minutes. Section were then incubated for 30 minutes in a solution containing 50 µg/ml of Oil Red O (Sigma - O0625, disolved in propylene glycol (Sigma – P4347)). Sections were then differentiated in 85% propylene gycol (in distilled water) for 1 minute and subsequently washed 3 times 1 minute in distilled water. Sections were finally cover slipped using Prolong Gold (Invitrogen, P36930) as mounting medium.

### Slide Imaging and Image Analysis

Sections were imaged at the McGill University imaging facility on a Zeiss Axiovert microscope (Zeiss, Germany). Frames for analysis were randomly sampled across each muscle section. Table 1 summarizes the number of fibers that were quantified for each muscle and for each species. For PGC-1α content, mitochondrial content and lipid content determination, individual fibers were traced using ImageJ software (NIH, USA). Once all the fibers were traced, the mean gray intensity for each fiber was determined, allowing the quantification of PGC-1α and mitochondrial content. For capillarization level determination, individual fibers on Lead-ATPase images were traced to obtain fiber perimeter. The capillary per fiber (C/F) ratio was then determined for each fiber. The C/F ratio was divided by the fiber perimeter of a given fiber to obtain the capillary to fiber perimeter exchange index (CFPE index) [20]. To determine the specific cytosolic PGC-1α content, a region of interest was demarcated by selecting a square (230 µm^2^) in the center of the fiber cytoplasm on the PGC-1α images using ImageJ, and the mean gray intensity corresponding to this region was computed. For the nuclear PGC-1α content determination, myofiber nuclei were traced using ImageJ on DAPI images and saved as regions of interest. These regions of interest were then transferred to the matching PGC-1α images. The mean gray intensity of these nuclear regions on the PGC-1α images was computed to determine the nuclear PGC-1α content.

**Table 1:** Average number of fibers analyzed per muscle and per species.

	PGC-1α content	Mitochondrial content	Capillarization	Lipid Content
**Mouse gastrocnemius**	434±65 (n = 6)	155±29 (n = 6)	ND	ND
**Rat plantaris**	176±42 (n = 6)	151±63 (n = 5)	63±16 (n = 4)	ND
**Rat Soleus**	189±28 (n = 6)	173±32 (n = 6)	57±19 (n = 4)	ND
**Human vastus lateralis**	168±97 (n = 6)	147±74 (n = 6)	ND	164±36 (n = 11)

Average number of fibers analyzed per muscle and per species for each of the parameters that was investigated (i.e. PGC-1 content, mitochondrial content, capillarization and lipid content). Data are expressed as mean ± SD. ND: Not Determined.

### Statistics

Values are presented as mean ± SEM. Details on statistical analyses performed for each set of data are provided in the legend of each figure.

## Results

### Specificity of the PGC-1α antibody

**Figure 1:**
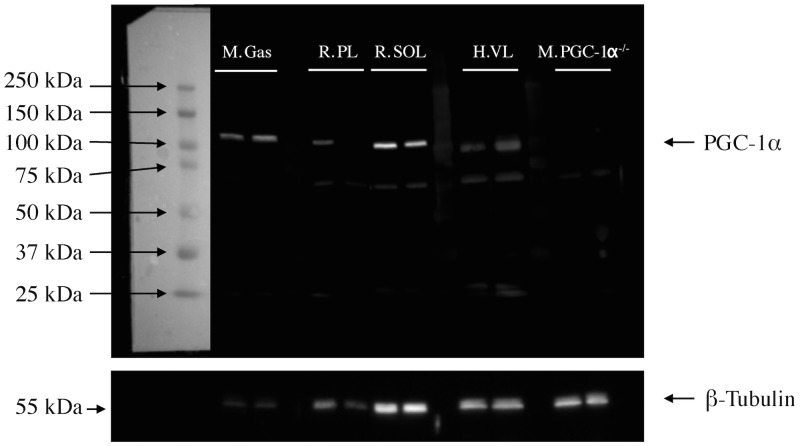
Verification of the specificity of the PGC-1α antibody. Representative images of PGC-1α (approx. 100 KDa) and β-tubulin (approx. 50 KDa - loading control) western blots performed in mouse gastrocniemius (M. Gas) rat soleus (R. SOL) and plantaris (R. PL) muscles and muscle homogenate obtained from PGC-1α^−/−^ mice (M. PGC-1α^−/−^).

To ensure the specificity of our PGC-1α antibody, we first determined PGC-1α content in homogenates of mouse gastrocnemius muscle, rat soleus and plantaris muscles, human vastus lateralis muscle and in a mixture of skeletal muscles obtained from PGC-1α knock out mice. As can be seen in Figure 1, an intense band slightly above 100 KDa (corresponding to the expected molecular weight of PGC-1α) was observed in all muscles except for skeletal muscles from PGC-1α knock out mice. While no band was observed for samples from PGC-1α knock out mice, it is important to note that the amount of proteins loaded for PGC-1α knock out mice was much higher as compared to the amount of proteins loaded for the gastrocnemius of wild type mice (as evidence by the β-Tubulin band). The absence of a PGC-1α band in one rat plantaris muscle can be explained by the fact that the amount of proteins that was loaded for this sample was probably too low to detect PGC-1α (as evidence by the β-Tubulin band). Only one weak unspecific band was observed at around 60 KDa, demonstrating a low potential for our antibody to bind many unspecific proteins in muscle cross-sections. In addition, we have previously reported that this PGC-1α antibody was able to detect the increase of PGC-1α expression induced by plasmid-mediated overexpression of PGC-1α in mouse skeletal muscle (see Figure 4 of [21]). Taken altogether, these results demonstrate the specificity of our antibody towards PGC-1α.

**Figure 4:**
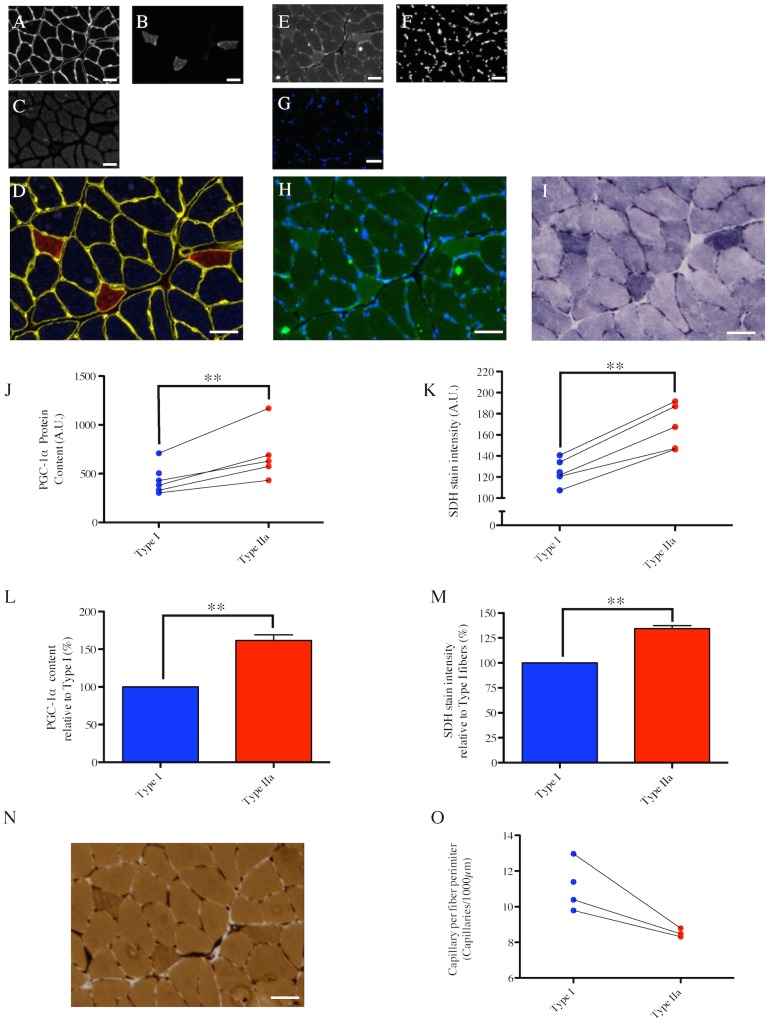
Fiber type-specific PGC-1α content in rat soleus muscle and its relation to mitochondrial content and capillarization. (A–C) in situ immunolabeling of a muscle cross-section for Myosin Heavy Chain (MHC) type IIb & laminin (A), type I (B), Type IIa (C). (D) Merge of MHC type IIb & laminin (yellow), type I (Blue) and type IIa (red) immunolabeling. (E–F) in situ immunolabeling for PGC-1α (E), and nuclei (F) obtained on a serial cross-section. A merge image of the PGC-1α and nuclei channels of a control cross-section where the PGC-1α primary antibody was omitted is presented in (G). (H) Merge of PGC-1α and nuclei immunolabelings. (I) in situ stain for Succinate DeHydrogenase activity (SDH) obtained on a serial cross-section. (J) Quantification of the fiber type-specific PGC-1α content (n = 6 rats, 189±28 fibers analyzed per animal). (K) Quantification of the fiber type-specific SDH stain intensity (n = 6 rats, 173±32 fibers analyzed per animal). Before being analyzed, SDH images were inverted in ImageJ for the quantified signal to be directly proportional to the SDH activity. (L) Fiber type-specific PGC-1α content relative to type I fibers. (M) Fiber type-specific SDH stain intensity relative to type I fibers. (N) Lead-ATPase stain performed on a serial section to visualize capillaries. (O) Quantification of the fiber type-specific capillary number per fiber perimeter (n = 4 rats, 57±19 fibers analyzed per animal). (K, L, P) Values arising from the same animal are connected by a line. (J–M) **: p<0.01. Statistical comparisons were performed using a paired two-tailed t-test. Scale bar: 50 µm.

### Fiber type-specific PGC-1α content and its relation to basal mitochondrial content in mice

**Figure 2:**
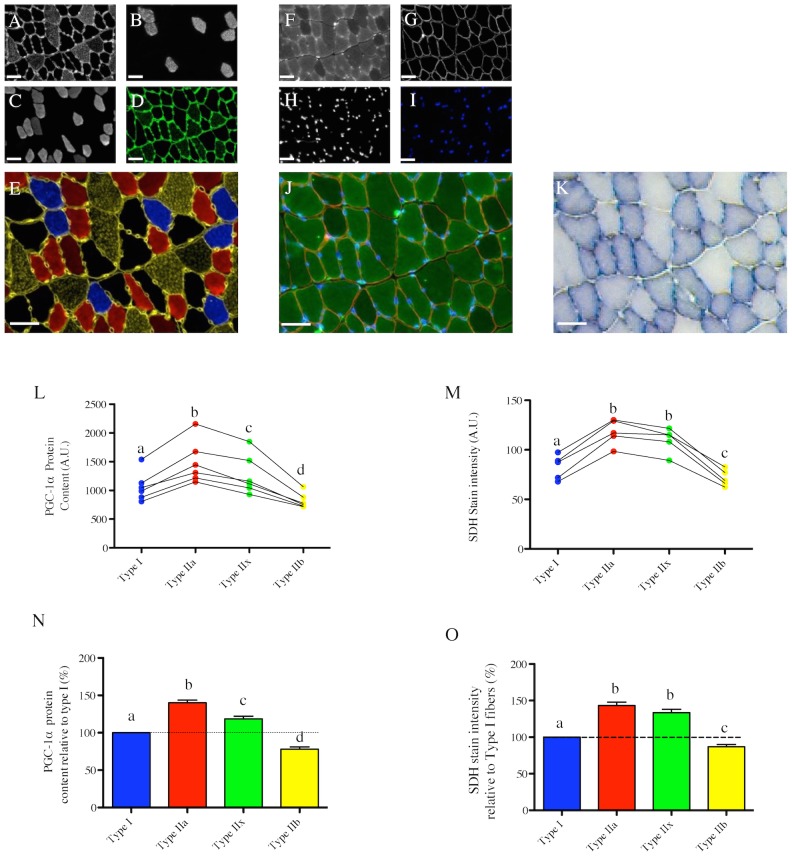
Fiber type-specific PGC-1α content in the mouse gastrocnemius muscle and its relation to mitochondrial content. (A–C) in situ immunolabeling of a muscle cross-section for Myosin Heavy Chain (MHC) type IIb & laminin (A), type I (B), Type IIa (C). (D) Immunolabeling for MHC type IIx (green) was performed on a serial cross-section. (E) Merge of MHC type IIb & laminin (yellow), type I (Blue) and type IIa (red) immunolabeling (type IIx fibers appear in black). (F–H) in situ immunolabeling for PGC-1α (F), dystrophin (G) and nuclei (H) obtained on a serial cross-section. A merged image of the PGC-1α, dystrophin and nuclei channels of a control cross-section where the PGC-1α and dystrophin primary antibodies were omitted is presented in (I). (J) Merge of PGC-1α, dystrophin and nuclei immunolabelings. (K) in situ stain for Succinate DeHydrogenase activity (SDH) obtained on a serial cross-section. (L) Quantification of the fiber type-specific PGC-1α content (n = 6 mice, 434±65 fibers analyzed per animal). (M) Quantification of the fiber type-specific SDH stain intensity (n = 5 mice, 155±29 fibers analyzed per animal). Before being analyzed, SDH images were inverted in ImageJ for the quantified signal to be directly proportional to the SDH activity. (N) Fiber type-specific PGC-1α content relative to type I fibers. (O) Fiber type-specific SDH stain intensity relative to type I fibers. (L, M) Values arising from the same animal are connected by a line. (L–O) Fiber types that do not share the same letter are significantly different (p<0.05). Statistical comparisons were performed using a one-way anova with repeated measures and a Tukey’s post hoc test. Scale bar: 50 µm.

Immunolabeling of serial cross sections of mouse gastrocnemius muscle were used in assessing fiber type (MHC immunolabeling) and PGC-1α content (Fig. 2A–J). Quantification of the fiber type-specific PGC-1α content revealed that type IIa fibers have the highest PGC-1α content in mouse gastrocnemius muscle, while type IIb fibers have the lowest (Fig. 2L and N). Interestingly, our results show that type IIx fibers have a higher PGC-1α than type I fibers (Fig. 2L and N). To assess mitochondrial content, sections serial to those used for MHC and PGC-1α immunolabeling were stained for succinate dehydrogenase (SDH) activity, a method that has been widely used to assess mitochondrial content in muscle cross sections [22]–[25] (Fig. 2K). Based on this SDH stain intensity, the fiber type with the highest mitochondrial content in mouse skeletal muscle is type IIa, followed by type IIx>type I>Type IIb (Fig. 2M and O). Although mitochondrial content between Type IIa and Type IIx was not significantly different, this absence of statistical difference is most probably explained by our small sample size, since 5 out of 6 animals displayed higher mitochondrial content in type IIa fibers as compared to type IIx (Fig. 2M and O). These results were further confirmed by the quantification TOM20 stain intensity, another reliable marker of mitochondrial content (Fig. S1A–B) [26], [27], which provided results similar to the one we obtained with the SDH stain.

### Fiber type-specific PGC-1α content and its relation to basal mitochondrial content and capillarization in rats

**Figure 3:**
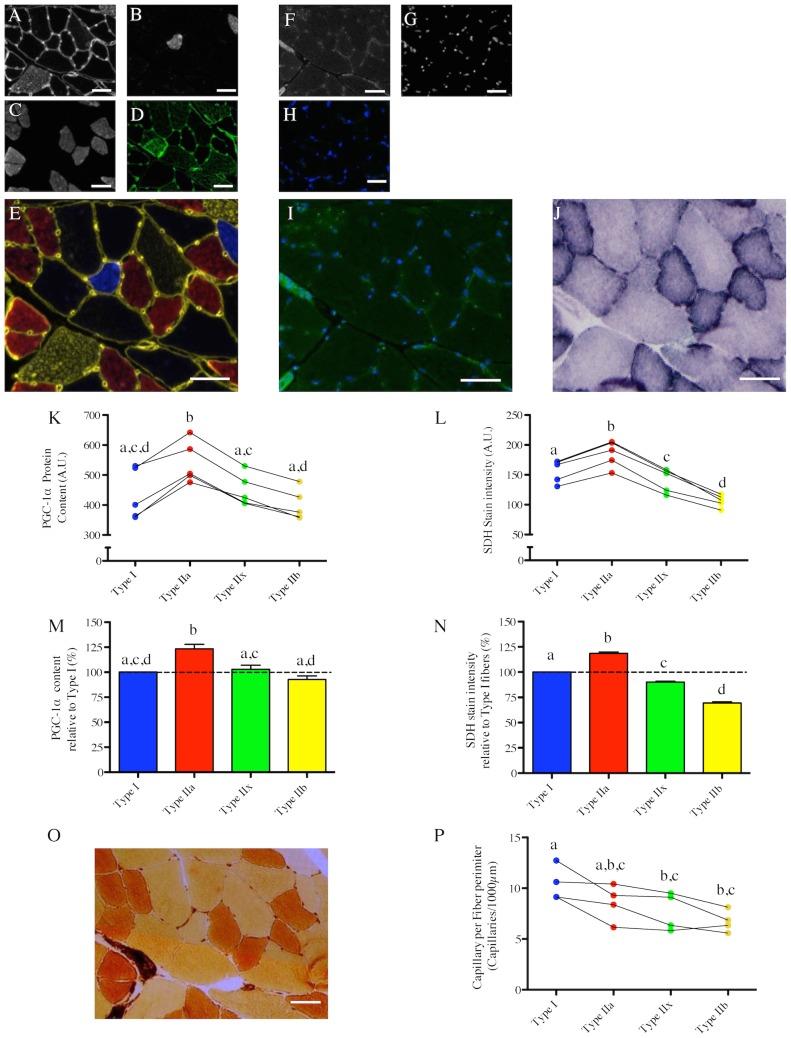
Fiber type-specific PGC-1α content in the rat Plantaris muscle and its relation to mitochondrial content and capillarization. (A–C) in situ immunolabeling of a muscle cross-section for Myosin Heavy Chain (MHC) type IIb & laminin (A), type I (B), Type IIa (C). (D) Immunolabeling for MHC type IIx (green) performed on a serial cross-section. (E) Merge of MHC type IIb & laminin (yellow), type I (Blue) and type IIa (red) immunolabeling (type IIx fibers appearing in black). (F–G) in situ immunolabeling for PGC-1α (F), and nuclei (G) obtained on a serial cross-section. A merge image of the PGC-1α and nuclei channels of a control cross-section where the PGC-1α primary antibody was omitted is presented in (H). (I) Merge of PGC-1α and nuclei immunolabelings. (J) in situ stain for Succinate DeHydrogenase activity (SDH) obtained on a serial cross-section. (K) Quantification of the fiber type-specific PGC-1α content (n = 5 rats, 176±42 fibers analyzed per animal). (L) Quantification of the fiber type-specific SDH stain intensity (n = 5 rats, 151±63 fibers analyzed per animal). Before being analyzed, SDH images were inverted in ImageJ for the quantified signal to be directly proportional to the SDH activity. (M) Fiber type-specific PGC-1α content relative to type I fibers. (N) Fiber type-specific SDH stain intensity relative to type I fibers. (O) Lead-ATPase stain performed on a serial section to visualize capillaries. (P) Quantification of the fiber type-specific capillary number per fiber perimeter (n = 4 rats, 63±16 fibers analyzed per animal). (K, L, P) Values arising from the same animal are connected by a line. (K, L, M, N, P) Fiber types that do not share the same letter are significantly different (p<0.05). Statistical comparisons were performed using a one-way anova with repeated measures and a Tukey’s post hoc test. Scale bar: 50 µm.

Fiber type, PGC-1α content, mitochondrial content and capillarization were determined in serial cross section of the rat plantaris (PL) and soleus (SOL) muscle (Fig. 3 and 4). In the PL, the fiber type with the highest PGC-1α content is type IIa, followed by type IIx = type I>type IIb (Fig. 3K and M). Although PGC-1α content in type IIb fibers was not significantly different from the type I PGC-1α content, all animals but one had PGC-1α content higher in type I as compared to type IIb fibers (Fig. 3K). In line with our results obtained in the PL, in SOL type IIa fibers have a greater PGC-1α content compared to type I fibers (Fig. 4J and L).

Quantification of the SDH staining intensity in the rat PL muscle (Fig. 3J) revealed that type IIa have the highest mitochondrial content followed by type I>type IIx>type IIb (Fig. 3L and N). These results were further confirmed by the quantification of the TOM20 stain intensity (Fig. S1E–H), which gave an identical result to that obtained by the quantification of SDH staining intensity. Similarly, mitochondrial content was higher in type IIa fibers as compared to type I fibers in the SOL (Fig. 4I, K and M).

Fiber type-specific capillarization levels were investigated using the Lead-ATPase stain and the determination of the CFPE index [20] in both rat PL (Fig. 3O) and SOL (Fig. 4N) muscles. As shown in Figure 3P, the fiber type with the highest CFPE index in PL muscle is type I, followed by type IIa>type IIx>type IIb. Similarly, type I fibers have a higher CFPE index as compared to type IIa in the SOL (Fig. 4O).

### Fiber type-specific PGC-1α content and its relation to basal mitochondrial and lipid contents in humans

**Figure 5:**
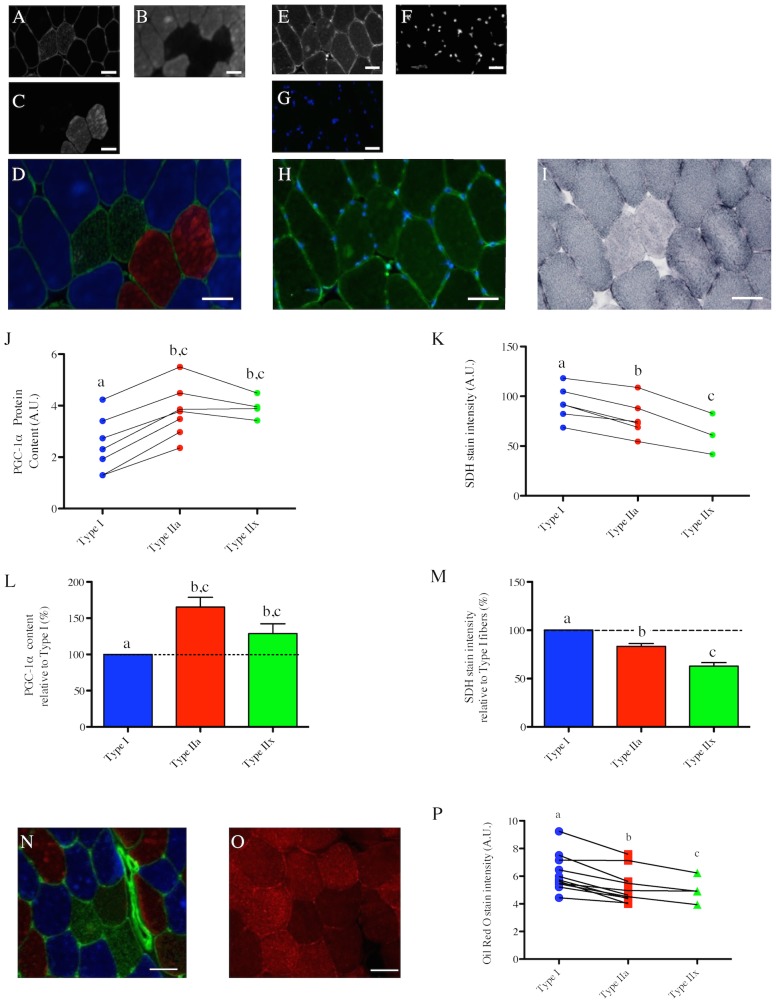
Fiber type-specific PGC-1α content in human vastus lateralis muscle and its relations to mitochondrial and lipid contents. (A–C) in situ immunolabeling of a muscle cross-section for Myosin Heavy Chain (MHC) type IIx & laminin (A), type I (B), Type IIa (C). (D) Merge of MHC type IIx & laminin (green), type I (Blue) and type IIa (red) immunolabeling. (E–F) in situ immunolabeling for PGC-1α (E), and nuclei (F) obtained on a serial cross-section. A merge image of the PGC-1α and nuclei channels of a control cross-section where the PGC-1α primary antibody was omitted is presented in (G). (H) Merge of PGC-1α and nuclei immunolabelings. (I) in situ stain for Succinate DeHydrogenase activity (SDH) obtained on a serial cross-section. (J) Quantification of the fiber type-specific PGC-1α content (n = 7 subjects, 168±97 fibers analyzed per subject). (K) Quantification of the fiber type-specific SDH stain intensity (n = 6 subjects, 147±74 fibers analyzed per subject). Before being analyzed, SDH images were inverted in ImageJ for the quantified signal to be directly proportional to the SDH activity. (J, K) Values arising from the same subject are connected by a line. (L) Fiber type-specific PGC-1α content relative to type I fibers. (M) Fiber type-specific SDH stain intensity relative to type I fibers. (J–M) Fiber types that do not share the same letter are significantly different (p<0.05). Due to the fact that no type IIx fibers were identified in 3 out of the 7 subjects, statistical comparisons were performed using paired two-tailed t-tests. (N–O) in situ immunolabeling of a muscle cross-section for MHC type IIx & laminin (green), type I (Blue) and type IIa (red) (N) and its corresponding Oil Red O stain (a marker of lipid content performed on a muscle serial cross-section (O). (P) Quantifications of the fiber type-specific Oil Red O stain intensity (N = 11). Fiber types that do not share the same letter are significantly different (p<0.05). Due to the fact that no type IIx fibers were identified in 4 out of the 11 subjects, statistical comparisons were performed using paired two-tailed t-tests. Scale bar: 50 µm.

Fiber type, PGC-1α content, mitochondrial content and lipid content were determined in serial cross sections of human vastus lateralis (VL) muscle (Fig. 5). In human VL muscle, the fiber type with the highest PGC-1α content was type IIa, followed by type I>IIx (Fig. 5J and L).

Quantification of SDH stain intensity revealed that type I fibers have the highest mitochondrial content in human VL, followed by type IIa>IIx (Fig. 5K and M), and this was confirmed by quantification of TOM20 and VDAC staining intensities (2 other markers of mitochondrial content) (Fig. S1I–O). Quantification of the Oil Red O (a marker of lipid content) stain intensity revealed that type I fibers have the highest lipid content in human VL, followed by type IIa>IIx (Fig. 5N–P).

### Fiber type-specific localization of PGC-1α

**Figure 6:**
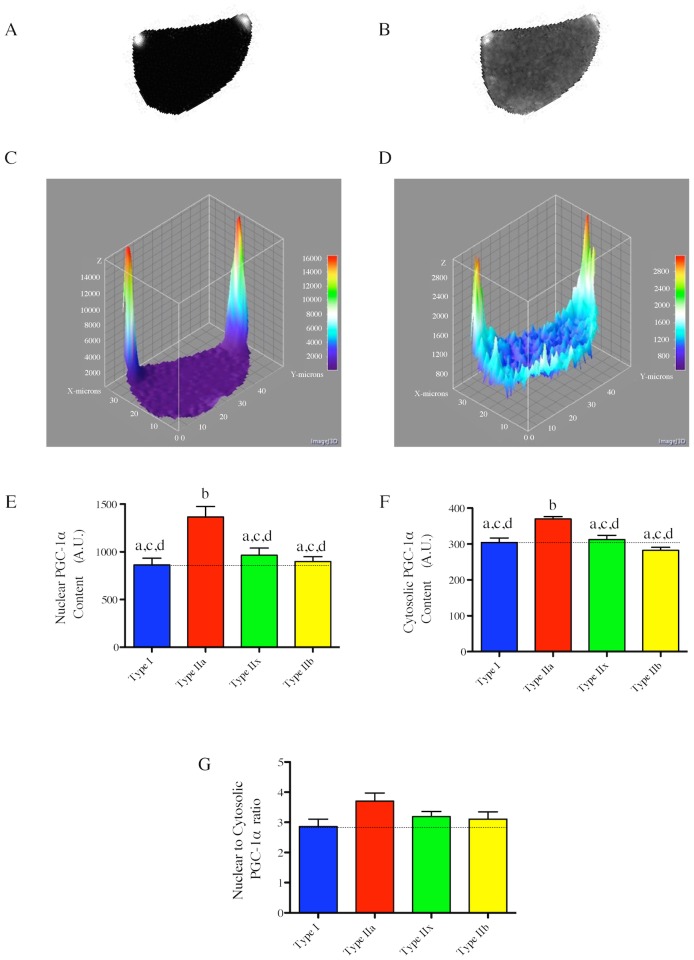
Fiber type-specific nuclear and cytosolic PGC-1α content in the rat plantaris muscle. Representative nuclei (A) and PGC-1α (B) immunolabeling of an individual fiber (traced and isolated using ImageJ). (C–D) Surface plot of nuclei (C) and PGC-1α (D) of the images shown in (A) and (B) respectively. (E–F) Quantifications of the fiber type-specific nuclear (E) and cytosolic (F) PGC-1α content (performed on 9 fibers for each fiber type). (G) Nuclear to cytosolic PGC-1α ratio. (E–F–G) Fiber types that do not share the same letter are significantly different (p<0.05). Statistical comparisons were performed using a one-way anova and a Tukey’s post hoc test.

To define whether different localization of PGC-1α (i.e. nuclear *vs* cytosolic) could exist between fiber types, we determined the fiber type-specific nuclear and cytosolic PGC-1α, as well as the ratio of nuclear *vs* cytosolic PGC-1α content, in rat PL muscle (Fig. 6). As can be seen in Figure 6E and F, the fiber type with the highest nuclear and cytosolic PGC-1α content is type IIa. Interestingly, no difference in nuclear PGC-1α content was detected between type I, IIx and IIb fibers. The pattern of the cytosolic PGC-1α content was almost identical as the one describe in Figure 3K and M. The determination of the nuclear vs cytosolic PGC-1α contents only revealed a trend for a higher value of this ratio in Type IIa fibers compared to the other fiber types (Fig. 6G).

## Discussion

Since its discovery in 1998 [28], multiple roles for PGC-1α in skeletal muscle physiology have been identified, principally using transgenic mice as a model system to manipulate PGC-1α levels. For example, skeletal muscle transgenic overexpression of PGC-1α drives the formation of type I fibers [1], increases mitochondrial content [1], [4], [5] and increases muscle capillarization in mouse skeletal muscle [9]. Whilst these genetic manipulations have undoubtedly been important to establish the role of PGC-1α in skeletal muscle biology, there have been no systematic comparisons of the results obtained in mice to other commonly employed animal models, nor has there been a comparison with human muscle. Furthermore, the relationships between PGC-1α and mitochondrial content at the whole muscle level, where oxidative muscle has both higher PGC-1α and higher mitochondrial content [1], [29], , have never been systematically analyzed at the individual fiber type level where there is a rich history of literature documenting a marked gradient in fiber mitochondrial content between subclasses of fast twitch fibers (see [31] for a detailed review). To address these issues our study examined the relationships between PGC-1α and mitochondrial content at the individual fiber level to examine the fiber type-specific relationships between these variables, and compared these relationships between two common rodent models (mouse and rat) and humans. Based on the experimental evidence in mice suggesting that PGC-1α drives the formation of type I fibers and regulates fiber mitochondrial content, lipid content and capillarization, we hypothesized (i) that PGC-1α content should be highest in type I fibers and (ii) that PGC-1α content should correlate with mitochondrial content, lipid content and anatomic capillarization.

In contrast to our first hypothesis, our data showed type IIa fibers, and not type I fibers, have the highest PGC-1α content in all three species that we investigated. This result strengthens and extends the work of Russell et al. who previously reported a higher PGC-1α content in human type IIa fibers using a similar experimental approach [32]. More surprising is the fact that type IIx fibers, usually considered as less oxidative than type I fibers in term of metabolic profile [33], [34], have either higher (mouse and human muscles) or similar (rat muscle) PGC-1α content compared to type I fibers. In this regard, Russell et al. reported that type IIx fibers had lower PGC-1α content compared to type I fibers in the vastus lateralis of sedentary human subjects; however, this difference between type I and IIx fibers disappeared after a 6-week endurance training program [32]. The fact that our human subjects were fairly active (147±42 min of physical activity per week) might therefore explain the discrepancy between our findings and those obtained by Russell et al. with regard to the type IIx PGC-1α content in their subjects prior to initiating exercise training. The higher PGC-1α content found in type IIa fibers in all species, associated with the similar (rat) or higher (mouse and human) PGC-1α in type IIx fibers versus type I fibers, therefore raises important questions about the role played by PGC-1α as a primary factor involved in determining the type I fiber phenotype [1]. Further to this point, it seems unlikely that an increase in PGC-1α with exercise training could be responsible for an increase in type I fiber abundance following adaptation [32] given that type IIa fibers already start with a higher PGC-1α content in mice, rats and humans. On the other hand, if, as our results suggest, each fiber type has its own baseline of PGC-1α content, a training-induced increase above this level even in a type IIa fiber could conceivably initiate a fiber type transition towards the type I phenotype. For this scenario to be valid, upon the transition from type IIa to type I fiber the PGC-1α content would need to decline to the new baseline that is ‘normal’ for the type I fiber. Further study will be required to determine if this sequence of events occurs. Notwithstanding this point, our results are also consistent with the emerging view that PGC-1α is only one of several important mechanisms that drive endurance exercise adaptation [35] and that there is significant modulation of PGC-1α signaling to fine-tune the muscle phenotype [36]. Other key regulators of myofiber phenotype, such as mitogen-activated protein kinase (MAPK) [37], calcineurin [38], calcium/calmodulin-dependent protein kinase (CaMK) [39], several isoforms of the nuclear factor of activated T-cells (NFATs) [35], and the nuclear receptor corepressor RIP140 [40] are some likely factors modulating the impact of PGC-1α content as a determinant of fiber type under basal conditions.

Amongst the roles played by PGC-1α in skeletal muscle physiology, its role in regulating mitochondrial biogenesis is the most established [1], [4], [5]. We therefore hypothesized that differences in PGC-1α content across fiber types should match differences in mitochondrial content. Consistent with this hypothesis, the fiber type-specific PGC-1α content paralleled the fiber type-specific mitochondrial content in mouse skeletal muscle, where the hierarchy for both factors was IIa>IIx>I>IIb. (Fig. 2), a result that is consistent with the previously established distribution of mitochondrial content across fiber types in mouse muscles [25]. On the other hand, in contrast to mouse skeletal muscle, no relationship between PGC-1α content and mitochondrial content was evident in rat PL or human VL muscles. In the rat PL muscle, although type IIx fibers had identical PGC-1α content as type I fibers, they displayed significantly lower mitochondrial content. Even more striking was the fact that human type IIa fibers, which have 65% more PGC-1α compared to type I fibers, had a 17% lower mitochondrial content. Further to this, human type IIx fibers, which show 28% more PGC-1α than type I fibers, have 37% lower mitochondrial content. These findings suggest that even though the role of PGC-1α in mediating mitochondrial biogenesis is well-established in mouse muscle, it is not the dominant determinant of basal mitochondrial content in rat or human skeletal muscle. Our results also support previous reports [25], [31], [41] in their conclusion that the fiber type-specific mitochondrial content pattern differs between species and that type IIa fibers, not type I fibers, have the highest mitochondrial content in rodent skeletal muscle, whereas type I fibers generally have greater mitochondrial content than type IIa fibers in human muscle. Finally, our results suggest that differences in PGC-1α content levels alone cannot explain fiber type-specific differences in mitochondrial content and that other regulators of mitochondrial biogenesis, such as peroxisome proliferator-activated receptor δ (PPARδ) [33], RIP140 [40] or nuclear receptor corepressor 1 (NCoR1) [42], [43] must be involved in regulating basal mitochondrial content. It should also be considered that since mitochondria are continually degraded and replaced [44], static levels of PGC-1α must play an important a role in determining rates of mitochondrial protein turnover. If turnover rates differ between fibers (e.g., perhaps it is higher in fast twitch muscle where mitochondrial ROS emission is known to be higher; [45]) and if this varies between species, this could be another important contributor to the variable correspondence between baseline levels of fiber type-specific mitochondrial content and PGC-1α levels between species.

Recent evidence suggests that PGC-1α might play an important role in the regulation of lipid droplet formation. Indeed, it was shown that PGC-1α overexpression in mice [10] or in cultured human skeletal muscle cells [11] leads to an increase in intramyocellular lipid droplet content. Based on these data, we hypothesized that the fiber type-specific PGC-1α content should relate to differences in intramyocellular lipid content. In contrast to this hypothesis, we found that the fiber type-specific PGC-1α strikingly differs from the distribution of intramyocellular lipid content across fiber types in human skeletal muscle. On the other hand, we found that the fiber type-specific lipid content in human skeletal muscle closely matches the distribution of mitochondrial content across fiber types, a result consistent with previously published experimental data [46]. Our results therefore suggest that differences in PGC-1α content alone cannot explain fiber type-specific differences in myocellular lipid content under basal conditions and indicate that other specific molecular mechanisms might play a prominent role in coordinating basal mitochondrial and lipid contents.

The last hypothesis we tested relates to the role of PGC-1α in the regulation of muscle capillarization. Indeed, it was clearly established that PGC-1α can regulate angiogenesis in transgenic mice with muscle-specific PGC-1α over-expression [9]. We therefore hypothesized that differences in PGC-1α content across fibers should relate to differences in capillarization levels. We found in rat PL muscle that despite the fact that PGC-1α content in type I fibers was lower than that of type IIa fibers and similar to type IIx fibers, type I fibers had the highest level of capillarization as indicated by CFPE index, a measure of capillarity that relates to the structural capacity for oxygen flux from capillary to muscle fiber [47]. Similarly, despite type I fibers in the rat SOL muscle having a lower PGC-1α content compared to type IIa fibers, they displayed higher CFPE index values indicating a higher level of capillarization. These disconnects between PGC-1α content and capillarization therefore challenge the role played by PGC-1α in determining basal capillarization level [9] and suggest that other factors might play a more prominent role and/or that there is significant modulation downstream of PGC-1α signaling in determining basal capillarization levels, for example, at the level of estrogen-related receptor alpha (ERRα). In addition, other signaling molecules, such as nitric oxide, hypoxia-inducible factor 1 (HIF-1) and signal transducer and activator of transcription 3 (STAT3) (see [48] for a detailed review) might play more prominent roles than PGC-1α in determining basal myofiber capillarization.

It could be argued that the total PGC-1α content might not be the most relevant parameter to investigate since it is well established that PGC-1α needs to undergo posttranslational modifications, such as phosphorylation and deacetylation, to be active [49]–[51]. Once phosphorylated and deacetylated, PGC-1α can translocate to the nucleus, where it exerts its activity [49]–[51]. Thus, to define whether differences in nuclear PGC-1α content could have explained disconnects between fiber type, total PGC-1α and mitochondrial content reported in the present study, we quantified the fiber type-specific nuclear content of PGC-1α in rat PL muscle. We found that the pattern of nuclear PGC-1α content across fiber types was similar to the one observed at the whole fiber level in that type IIa fibers had the highest nuclear PGC-1α content. No difference in the nuclear to cytosolic PGC-1α ratio was observed across fiber types. These results therefore indicate that differences in nuclear PGC-1α content across fiber types are unlikely to explain the disconnects we observed between fiber type, total PGC-1α content, mitochondrial content and capillarization level at the individual fiber level.

Recent studies have demonstrated the existence of splice variants of PGC-1α that are expressed in skeletal muscle, such as NT-PGC-1α and PGC-1α4 [52]–[54]. Given the fact that PGC-1α immunolabeling performed in the present study were obtained using an antibody that recognizes the C-terminus of the full-length PGC-1α protein, it is possible that some truncated PGC-1α splice variants were not detected. However, given available evidence in the literature, it seems very unlikely that the best characterized truncated PGC-1α splice variants, i.e. NT-PGC-1α and PGC-1α4, would play a key role in determining basal mitochondrial content, lipid content, fiber type and capillarization. Indeed, massive overexpression (60-fold increase) of NT-PGC-1α in mouse skeletal muscle had virtually no effect on the expression of genes involved in mitochondrial oxidative phosphorylation [55]. Although, massive NT-PGC-1α overexpression increases skeletal muscle capillarization in mice [55], it is important to bear in mind that NT-PGC-1α is predominantly located in the cytoplasm under basal conditions in skeletal muscle cells (i.e., almost undetectable in nuclei) [56], where it cannot exert its co-activator of transcription activity. In regards to a PGC-1α4, its overexpression has little to no effect on the expression of genes related to mitochondrial biology or angiogenesis [54]. Further to this point, it is important to keep in mind that the roles and functions of PGC-1α splice variants still remain controversial [53], [54], [57]. Further studies are therefore warranted to better define the roles played by PGC-1α splice variants in skeletal muscle physiology.

## Conclusions

The results of present study suggest that the dominant contribution of PGC-1α in promoting aerobic phenotypes such as mitochondrial and lipid contents, type I fibers, and capillarization at the single fiber level, at least under basal conditions, might have been overestimated. Although transgenic PGC-1α overexpression was shown to increase the abundance of type I fibers in mice [1], we consistently found across species that type IIa fibers have the highest PGC-1α content. Furthermore, in humans, PGC-1α content was actually lowest in type I fibers despite these fibers having the highest mitochondrial content. These results therefore challenge the importance of PGC-1α in determining the type I fiber phenotype under normal (non-transgenic) physiological circumstances. In addition, although the fiber type-specific PGC-1α content paralleled differences in mitochondrial content in mouse skeletal muscle, results obtained in rat and human skeletal muscles demonstrated several disconnects between fiber type, PGC-1α content, mitochondrial content, lipid content and capillarization levels. Taken altogether, our results suggest that PGC-1α is unlikely to be the most important determinant of fiber type, mitochondrial content, lipid content and capillarization under normal physiological conditions, a view in line with recent findings indicating that PGC-1α and β are dispensable for baseline mitochondrial content and fiber typing [58]. Future studies should therefore not overlook other important modulatory factors (such as CaMK, calcineurin, NFATs, MAPK, PPARδ, RIP140, NCoR1, HIF-1, STAT3) and seek to further understand how PGC-1α’s effects are modulated in regulating these key phenotypes in skeletal muscle under physiologically relevant conditions. In addition, our results highlight important differences in the fiber-type-specific PGC-1α and mitochondrial contents across species. In particular, these fiber type-specific contents differ largely between mouse and human skeletal muscles, with rat skeletal muscles showing intermediate properties. Such differences might be explained by the fact that the rat genome appears, in several respects, intermediate between the mouse and human genome [59]–[61]. Our results therefore warrant caution in extrapolating results obtained in rodent skeletal muscles, and especially in mouse, to human skeletal muscle.

## Supporting Information

Figure S1 **Quantification of the fiber type specific mitochondrial in mouse, rat and human skeletal muscles using different marker of mitochondrial content.** (A–B) in situ immunolabeling of a mouse gatrocnemius cross-section for MHC type IIb & laminin (yellow), type I (Blue) and type IIa (red) (type IIx fibers appear in black) (A) and its corresponding TOM20 (a marker of mitochondrial content; in red) & dystrophin (green) immunolabeling performed on a serial cross-section (B). (C) Quantifications of the fiber type-specific TOM20 stain intensity in mouse gastrocnemius muscle (N = 4). (D) Fiber type-specific TOM20 stain intensity relative to type I fibers in mouse gastrocnemius muscle. (E–F) in situ immunolabeling of a rat plantaris cross-section for MHC type IIb (yellow), type I (Blue) and type IIa & laminin (red) (type IIx fibers appear in black) (E) and its corresponding TOM20 immunolabeling (red) & dystrophin (green) performed on a serial cross-section (F). (G) Quantifications of the fiber type-specific TOM20 stain intensity in rat plantaris muscle (N = 3). (H) Fiber type-specific TOM20 stain intensity relative to type I fibers in rat plantaris muscle. (I–J) in situ immunolabeling of a human vastus lateralis cross-section for MHC type IIx & laminin (green), type I (Blue) and type IIa (red) (I) and its corresponding TOM20 (red) & dystrophin (green) immunolabeling performed on a serial cross-section (J). (K) Quantifications of the fiber type-specific TOM20 stain intensity in human vastus lateralis muscle (N = 3). (L) Fiber type-specific TOM20 stain intensity relative to type I fibers in human vastus lateralis muscle. (M–N) in situ immunolabeling of a human vastus lateralis cross-section for MHC type IIx & laminin (green), type I (Blue) and type IIa (red) (M) and its corresponding VDAC (a marker of mitochondrial content) immunolabeling performed on a serial cross-section (N). (O) Quantifications of the fiber type-specific VDAC stain intensity in human vastus lateralis muscle. Results in (O) were obtained by quantifying the VDAC stain intensity in 44 fibers from 1 human vastus lateralis sample. Fiber types that do not share the same letter in (O) are significantly different (p<0.05). Statistical comparisons presented in (O) were performed using paired two-tailed t-tests. Scale bar: 100 µm.
(TIF)
