# Supplementary figures and images for: The Relationship between Muscle Fiber Type-Specific PGC-1α Content and Mitochondrial Content Varies between Rodent Models and Humans
Source: PLoS One. 2014 Aug 14;9(8):e103044. doi: 10.1371/journal.pone.0103044 (PMC4133187; doi:10.1371/journal.pone.0103044)

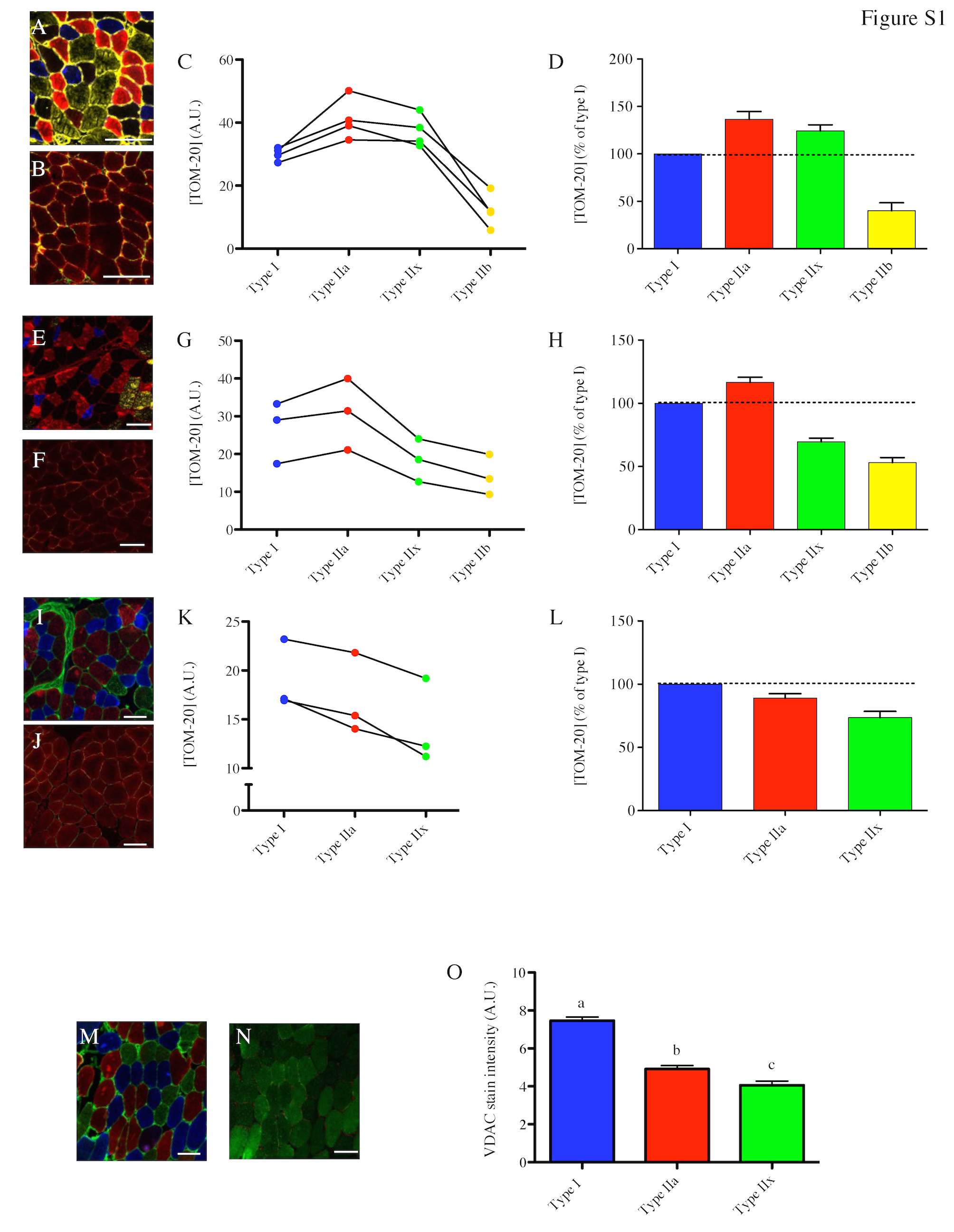

Supplement: Figure S1 — Quantification of the fiber type specific mitochondrial in mouse, rat and human skeletal muscles using different marker of mitochondrial content. (A–B) in situ immunolabeling of a mouse gatrocnemius cross-section for MHC type IIb & laminin (yellow), type I (Blue) and type IIa (red) (type IIx fibers appear in black) (A) and its corresponding TOM20 (a marker of mitochondrial content; in red) & dystrophin (green) immunolabeling performed on a serial cross-section (B). (C) Quantifications of the fiber type-specific TOM20 stain intensity in mouse gastrocnemius muscle (N = 4). (D) Fiber type-specific TOM20 stain intensity relative to type I fibers in mouse gastrocnemius muscle. (E–F) in situ immunolabeling of a rat plantaris cross-section for MHC type IIb (yellow), type I (Blue) and type IIa & laminin (red) (type IIx fibers appear in black) (E) and its corresponding TOM20 immunolabeling (red) & dystrophin (green) performed on a serial cross-section (F). (G) Quantifications of the fiber type-specific TOM20 stain intensity in rat plantaris muscle (N = 3). (H) Fiber type-specific TOM20 stain intensity relative to type I fibers in rat plantaris muscle. (I–J) in situ immunolabeling of a human vastus lateralis cross-section for MHC type IIx & laminin (green), type I (Blue) and type IIa (red) (I) and its corresponding TOM20 (red) & dystrophin (green) immunolabeling performed on a serial cross-section (J). (K) Quantifications of the fiber type-specific TOM20 stain intensity in human vastus lateralis muscle (N = 3). (L) Fiber type-specific TOM20 stain intensity relative to type I fibers in human vastus lateralis muscle. (M–N) in situ immunolabeling of a human vastus lateralis cross-section for MHC type IIx & laminin (green), type I (Blue) and type IIa (red) (M) and its corresponding VDAC (a marker of mitochondrial content) immunolabeling performed on a serial cross-section (N). (O) Quantifications of the fiber type-specific VDAC stain intensity in human vastus lateralis muscle. Re [file pone.0103044.s001.tif]
